# Exo-miRNAs as a New Tool for Liquid Biopsy in Lung Cancer

**DOI:** 10.3390/cancers11060888

**Published:** 2019-06-25

**Authors:** Orazio Fortunato, Patrizia Gasparini, Mattia Boeri, Gabriella Sozzi

**Affiliations:** Tumor Genomics Unit, Department of Research, Fondazione IRCCS Istituto Nazionale dei Tumori, via Venezian 1, 20133 Milan, Italy; mattia.boeri@istitutotumori.mi.it (M.B.); gabriella.sozzi@istitutotumori.mi.it (G.S.)

**Keywords:** microRNA, lung cancer, exosomes

## Abstract

Lung cancer is the predominant cause of cancer-related deaths. The high mortality rates are mainly due to the lack of diagnosis before the cancer is at a late stage. Liquid biopsy is a promising technique that could allow early diagnosis of lung cancer and better treatment selection for patients. Cell-free microRNAs have been detected in biological fluids, such as serum and plasma, and are considered interesting biomarkers for lung cancer screening and detection. Exosomes are nanovesicles of 30–150 nm and can be released by different cell types within the tumor microenvironment. Their exosomal composition reflects that of their parental cells and could be potentially useful as a biomarker for lung cancer diagnosis. This review summarizes the state-of-the-art of circulating microRNAs (miRNAs) in lung cancer, focusing on their potential use in clinical practice. Moreover, we describe the importance of exosomal miRNA cargo in lung cancer detection and their potential role during lung carcinogenesis. Finally, we discuss our experience with the analysis of circulating exosomal miRNAs in the bioMILD screening trial.

## 1. Introduction

Lung cancer is a leading cause of cancer deaths globally for both men and women, accounting for more than 1.4 million deaths per year [1]. In 2018, lung carcinoma was responsible for 14% of all new cancer diagnoses and about 25% of all cancer deaths [2]. Characterized by two major histotypes, lung cancer is mainly represented by non-small-cell lung cancer (NSCLC, about 80%) and by a smaller component of small-cell lung cancer (about 20%) [3]. In particular, in Italy, the estimated incidence of NSCLC is about 41,000 new cases per year [4]. Cigarette smoking is considered the number one risk factor for lung cancer. While anyone can develop lung cancer, cigarette smoking and exposure to smoke can increase the probability that a person will be affected by this condition [5]. Several factors influence the poor prognosis of this malignancy: absence of symptoms in early stages, limited understanding of lung cancer biology, tumor heterogeneity, lack of effective therapeutic strategies, and, most importantly, diagnosis at advanced stages [6]. To date, surgical intervention remains the preferred treatment for early-stage tumors, with chemotherapy regimens for advanced lung cancer patients, or a solely chemo-immunotherapeutic approach when the disease is metastatic [7]. Survival for lung malignancies drops drastically as the stage at diagnosis becomes more advanced.

Identification of driver mutations and genetic rearrangements in approximately 50–60% of NSCLC has led to a change in the treatment of lung cancer patients [8], by classifying subgroups of patients by different molecular profile. K-RAS mutations have been observed in approximately 17% of all NSCLC, in particular in adenocarcinomas (27–34%), whereas the discovery of activating mutations in the EGFR gene (23%) and rearrangements of anaplastic lymphoma (ALK) (5%) also demonstrated a relevant impact in the treatment of lung cancer patient, with their impressive response to tyrosine kinase inhibitor (TKI) agents, such as erlotinib, crizotinib and gefitinib [9]. Initially, tumor inhibition is reached through target therapies, but their efficacy in the majority of patients is limited by the onset of resistance mechanisms [3]. Moreover, for individuals with inoperable disease, outcomes are worse in those with poor performance status and a substantial weight loss of more than 10% [10]. If lung cancer is found at an earlier stage, this often allows for more treatment options and better overall survival. Despite improvements in early diagnosis and novel therapeutic interventions, the overall 5-year survival rate remains at only 10–20% [1]. Improving survival with screening tests and/or exams is a clinical need, and investigating molecular mechanisms essential for lung cancer development and progression is of critical importance.

Lung cancer development and progression is strongly regulated by the complex interplay between a tumor and its microenvironment, which can include stromal cells such as fibroblast, endothelial, and immune cells [11,12] as well as biomolecules, such as several types of growth and inflammatory factors, and proteases [13]. Mechanisms related to the tumor microenvironment, such as hypoxia, inflammation, angiogenesis, and exosomes, play pivotal roles in tumor development, invasion and metastasis [14]. It has been described that cancer cells could reprogram stromal cells to support carcinogenesis [15]. Particularly, lung cancer progression is driven not only by a tumor’s underlying genetic alterations but is also mediated by complex systemic interactions between cells in the tumor microenvironment by way of several mechanisms: cell–cell contacts (receptor-mediated interaction, gap junctions) or paracrine signals (growth factors, cytokines, and chemokines), as well as by extracellular vesicles (EVs), including exosomes [16]. Originally described as cellular garbage to eliminate excessive proteins or undesirable molecules from cells, exosomes are small (30–100 nm) membrane vesicles of endocytic origin which are actively secreted by most cell types, including cancer cells [17]. Recent knowledge revealed that different cell types constitutively release exosomes in order to mediate cell-to-cell communication both in normal and pathological states. Similarly to cells, exosomes contain an heterogeneous composition of biomolecules, such as lipids, proteins and nucleic acids (DNA, mRNA, non-coding RNA) that can be transversely delivered to recipient cells [18]. Several authors reported that tumor-derived cells could influence cells of the tumor microenvironment inducing EMT and extracellular matrix degradation [19]; modify endothelium permeability and activation [20]; affect immune cells status, especially exosomes isolated from circulating tumor cells [21] and finally altering microenvironment at distant sites to promote a suitable pre-metastatic niche [22].

miRNAs are short (19–24 nt), single-stranded non-coding RNAs that regulate gene expression at the post-transcriptional level, either by promoting the cleavage of target mRNAs or by repressing their translation [23,24]. miRNA biogenesis is tightly controlled, and their deregulation is associated to cancer [25]. In addition, miRNAs’ function as signaling molecules, influencing the behavior of recipient cells. Therefore, the detection of miRNAs in biological fluids, such as plasma or serum, could serve as crucial circulating biomarkers [26,27].

Recently, several studies have demonstrated the potential of exosomal-miRNAs in mediating several pathways involved in tumorigenesis. Breast cancer cells were observed to secrete exosomes with the potential to perform cell-independent miRNA biogenesis and to stimulate non-tumorigenic mammary epithelial cells to form tumors [28]. Further evidence has demonstrated that microvesicles secreted by macrophages transport miR-223 into breast cancer cells and were capable of promoting breast cancer invasiveness [29], that exosomes which originated from fibroblasts actively participated in the migration of breast cancer cells [30] and, finally, that exosomes from cancer cells held protumorigenic potential [31]. An immunomodulatory role for miRNAs transferred by extracellular vesicles was also reported [32]. However, the functional role of miRNAs associated with exosomes in lung cancer development and progression is mostly unknown, and a single study reported that miRNAs in cancer-secreted exosomes may act as paracrine agonists of Toll-like receptors, which are able to trigger a prometastatic inflammatory response in a Lewis lung carcinoma model [33].

This review is intended to summarize the potential role of exosomes-miRNAs for liquid biopsy in NSCLC detection. We first focus here on elucidating the potential role of circulating miRNAs in lung cancer clinical practice, as reported by critical studies. Then, we address the prospective use of exosomes as diagnostic and/or prognostic biomarkers and describing their function as active players in lung carcinogenesis. Finally, we illustrate our data, obtained by investigating exosomes and their miRNA cargo in bioMILD screening trials, consolidating their important impact in detection, definition, diagnosis, and clinical management in lung malignancies.

## 2. Circulating miRNA as a Biomarker for NSCLC

Analyzing miRNAs that are circulating in liquid biopsies, such as plasma and/or serum, could be the basis of a valid test which is able to screen biomarkers to discriminate NSCLC patients from healthy individuals. As summarized by Table 1, several studies to date have investigated the possible role of circulating miRNAs as potential biomarkers in NSCLC patients compared to healthy controls, utilizing high-throughput technologies [34,35].

In particular, the increased expression of miR-29 and reduction of 7 miRNAs (miR-146b, miR-221, let-7a, miR-155, miR-17-5p, miR-27a, and miR-106a) in the serum of early-stage NSCLC patients was observed by using real-time PCR [36]. This work also revealed that the circulating miRNAs levels in serum did not correlate with plasma from the same patients, suggesting different miRNA circulating compositions according to the blood component analyzed. Thus, a set of plasma miRNAs (miR-21, miR-126, miR-210, and miR-486-5p) were used as diagnostic biomarkers to distinguish NSCLC from controls, demonstrating an 86.2% sensitivity and 96.6% specificity, while increasing the incidence of histological diagnosis of adenocarcinoma compared to squamous cell carcinomas [37]. Three of these miRNAs were also successfully tested to discriminate malignant from benign lesions [44]. Also, a combination of circulating miR-155, miR-197, and miR-182 was able to differentiate stage I lung cancer patients from cancer-free individuals with good sensitivity (81.33%) and specificity (86.76%), revealing the potential of this analysis [38]. Moreover, a panel of miRNAs (miR-483, miR-193a-3p, miR-25, miR-214, and miR-7) was found to be significantly elevated in NSCLC patients compared to controls in a multicenter study of a cohort of Chinese and American individuals, strongly suggesting the remarkable diagnostic potential of these biomarkers for patients of different ethnicities [39]. All these studies highlighted the usefulness of circulating miRNAs as biomarker for the detection of advanced lung cancers.

As detecting lung cancer at early stages is critical for guiding clinical management and overall outcomes, circulating miRNAs are also investigated for their ability to detect early protumorigenic changes in high-risk individuals [45]. In this regard, using plasma samples collected from a pilot low-dose computer tomography (LDCT) lung cancer screening trial, our group identified 4 plasma-based miRNA signatures composed of reciprocal ratios of 24 miRNAs with both diagnostic and prognostic value [46]. This particular miRNA Signature Classifier (MSC) was subsequently validated in a large cohort of 1000 consecutive plasma samples from 4099 participants cancer patients enrolled in the MILD study [47], showing significant diagnostic performance with 87% sensitivity and 81% specificity [40]. On the basis of these results, our group proposed a large prospective study in heavy-smoker volunteers, the bioMILD trial, based on plasma miRNA profiling, in order to confirm its efficacy as a screening test for lung cancer detection when combined with LDCT [48]. On the other hand, Bianchi et al. investigated the potential role of circulating miRNAs in the serum of high-risk individuals. Interestingly, a panel of 34-miRNAs (miR-test) was able to classify asymptomatic high-risk individuals with early lung cancer and also discriminate malignant from benign nodules detected by LDCT screening. In particular, for high-risk lung cancer patients, the miR-test exhibits a diagnostic accuracy, sensitivity, and specificity of 74.9%, 77.8%, and 74.8%, respectively [49]. Furthermore, a reduced miR-test of 13 miRNAs was validated in the COSMOS (Continuous Observation of Smoking Subjects) lung cancer screening trial and in lung cancer patients diagnosed outside the screening [41], revealing a similar performance as in the study by Bianchi et al. [49]. Finally, another combination of 24 circulating plasma miRNAs was identified in a cohort of 100 early-stage (I–III) lung cancer patients and controls, and identified cases with an area under the curve (AUC) of 0.92 [42]. Interestingly, 6 miRNAs (miR-30b, miR-30c, miR-92, miR-140-5p, miR-145, and miR-148a) were identified to be recurrently present among the MSC and miR-test groups, and their importance in lung cancer detection should be explored further.

Recently, a systematic meta-analysis including 134 lung cancer studies, with a total of 6919 patients with lung cancer and 7064 controls, revealed the diagnostic significance of circulating miRNAs in lung cancer with a sensitivity of 83%, a specificity of 84%, and an AUC of 0.90. Additionally, circulating miRNAs demonstrated a higher diagnostic performance in the Caucasian population, while serum was considered a more suitable specimen for this type of analysis. Strikingly, the performance of circulating miRNAs in the detection of early lung cancer is quite high, with 81% sensitivity and 82% specificity for stage I–II cancer. Finally, miR-21, miR-223, miR-155, and miR-126 emerged as promising biomarkers for lung cancer detection [43].

However, the application of circulating miRNAs in the clinical routine has been hampered by several reasons mostly related to the heterogeneity of the studies, their limited sample size, the lack of prospective studies and large external validation. The lack of reproducibility of the results is largely attributable to pre-analytical factors (hemolysis, RNA isolation, data normalization), different technological platforms (RNAseq, qPCR, microarrays) and statistical approaches [50]. All these works provide evidence for the impact and potential that miRNAs can offer as liquid biopsy-based biomarkers for more accurate early lung cancer detection, and to ultimately guide the clinical management of this otherwise fatal disease.

## 3. Functional Role of Exosomal miRNAs in Lung Carcinogenesis

**Figure 1:**
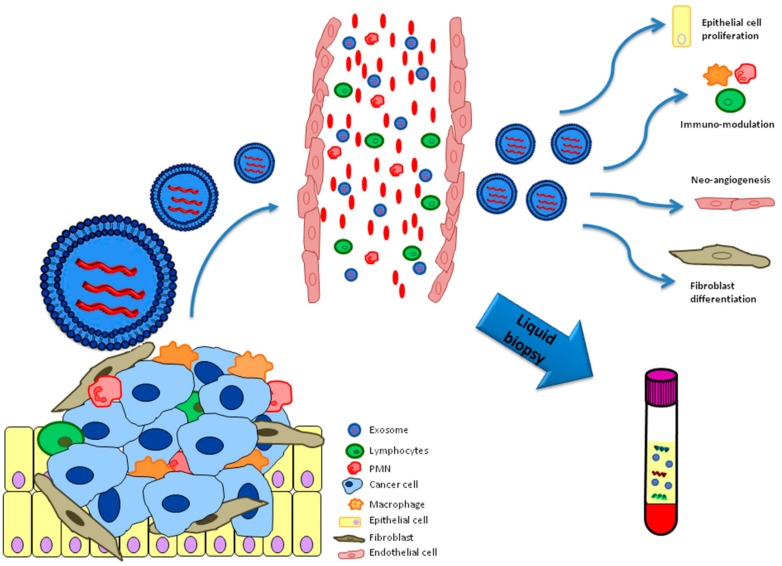
Functional role of exosomes released by the lung cancer microenvironment.

Cells possibly use exosomes as an intermediary for shuttling biomolecules to neighboring or distant cells and influencing their functionalities (Figure 1).

Recent evidence shows that exosomes are closely related to lung carcinogenesis and that tumor-derived exosomes play a crucial role in the growth and progression of lung malignancies by the modulation of a wide range of pathways, including tumor angiogenesis and EMT (epithelial mesenchymal transition) (Table 2) [51].

Furthermore, it has been demonstrated that exosomal miR-23a, overexpressed in hypoxic lung cancer, enhanced angiogenesis and vascular permeability through its targets prolyl hydroxylase (PHD) and tight junction protein-1 (also known as zonula occludens-1, ZO-1) [52]. In addition, hypoxia increased miR-103a levels in the exosomes of lung cancer cells and patients, modifying M2 macrophage phenotype through *AKT* and *STAT3* activation with a stimulatory effect on tumor progression and angiogenesis [21]. The specific increase of miR-210 levels in exosomes released from lung cancer cells was observed by the tissue inhibitor of metalloproteinase-1. Interestingly, these exosomes promoted tube formation activity in endothelial cells in vitro, also increasing angiogenesis and tumor progression in in vivo mouse xenograft models [53]. Rana et al. described how tumor-derived exosomes could transfer miRNAs in order to influence selected lymph nodes and eventually modulate pre-metastatic organ cells. Transgenic rat models of lung adenocarcinoma were utilized to demonstrate that the relocation of miR-494 and miR-542-3p from exosomes to stromal cells downmodulated cadherin-17 and induced pre-metastatic niche formation [54]. Another interesting aspect of tumor-suppressive miRNAs is that they could be silenced in tumor cells through epigenetic mechanisms. In this regard, the expression of several miRNAs was induced and these miRNAs were secreted in exosomes by tumor cells following epigenetic treatment using 5’ AZA and the addition of trichostatin A. In particular, tumor-derived exo-miR-512 and miR-373 were both associated with cisplatin sensitivity and the suppression of tumor progression in cultured lung cancer cells through the direct downmodulation of *TEAD4*, *RelA*, and *PIK3CA* [55]. Berchem et al. presented results showing that vesicles released by hypoxic lung cancer cells were able to inhibit NK cell function through two different mechanisms: one involving the transfer of TFG-B in recipient cells with the inhibition of activating surface receptor NKG2D, and the other through shifting miR-23, which targets CD107 expression in NK cells [56]. Also, miR-192 was shown to repress pro-angiogenic factors such as IL-8, ICAM, and CXCL1 when transferred from lung cancer cell-derived exosomes to endothelial cells. Thus, miR-192 impaired angiogenesis and reduced bone metastasis in in vivo models [60]. Another demonstration was given by tumor suppressor miR-302b, found in exosomes from low metastatic lung cancer cells, which is capable of blocking the proliferation and migration of highly metastatic lung cancer cells through the regulation of TGFBR II [57]. Finally, Zhuang et al. detected tumor-derived exosomes containing high levels of miR-9, which usually promotes endothelial cell migration and tumor angiogenesis in vivo. Exosomal miR-9 revealed the potential of activating the JAK-STAT pathway through the downmodulation of the suppressor of cytokine signaling 5 (SOCS5), a negative regulator of this specific pathway [58].

Although the bulk of the literature has concentrated on investigating the role of exo-miRNAs released by cancer cells, a few recent studies describe the detection of exo-RNAs derived from epithelial and stromal cells. Epithelial cells could be considered as a major producer of exosomes in the lung, contributing to lung homeostasis and lung cancer pathogenesis [61]. Strikingly, a study reported that cigarette smoking induced the secretion of exosomes containing miR-21 from bronchial cells, enhancing VEGF levels through STAT3 deregulation in Human Bronchial Epithelial Cells (HBEC) and promoting angiogenesis and tumor growth [59]. In addition, bronchial epithelial cells secrete miR-201, which promotes fibroblast differentiation and the autophagy mechanism after cigarette smoking [62]. The functional role of exosomes released by non-epithelial cells in the development of lung cancer has been investigated by a several research groups. Of particular importance are the results of one study which detected exosomal miR-223 being released by platelets, further demonstrating its involvement in lung cancer progression by modulating tumor cell invasion through the suppression of EPB41L3 [63].

These presented works show that exosomal-miRNAs reflects miRNAs expression patterns of the cell from which they derive from, suggesting an active release of these molecules by tumor cells for the modulation of lung microenvironment. However, the patho-physiological roles of extracellular vesicles remain unclear. To this regard, phenotype characterization of vesicles, sorting mechanisms for the content into exosomes, secretion mechanisms into the blood and the uptake system of recipient cells has not been fully investigated in any of these studies. Also, use of in vitro approaches alone, together with a small size of analyzed clinical samples, create limitation and weaknesses to these studies. Understanding the functional role of exosomes in cell to cell communication and in particular in the onset of the disease, are crucial points that need to be addressed.

## 4. Exosomal miRNA for NSCLC Detection

One of the most significant and attractive aspects of exosomes, including their genetic material, is their diagnostic and/or prognostic potential for several malignancies, in particular lung cancer, which was proposed and reinforced by several studies (Table 3).

A signature of 12 miRNAs (miR-17-3p, miR-21, miR-106a, miR-146, miR-155, miR-191, miR-192, miR-203, miR-205, miR-210, miR-212, and miR-214) assessed in a small group of NSCLC patients, were strictly correlated with miRNA concentrations observed in circulating exosomes and demonstrated their diagnostic impact in reflecting the tumor’s genetic profile. Surprisingly, the miRNA profiles did not correlate with the disease stage, leading to questions regarding their complete significance as a diagnostic marker [64]. Validation of the expression of 5 miRNAs (let-7f, miR-20b, miR-30e-3p, miR-223, and miR-301) in plasma exosomes reinforced their possible application in clinical settings in order to analyze and predict lung cancers in a non-invasive manner [65]. Cazzoli et al. identified two different miRNA signatures from exosomes derived from lung cancer patients: a screening one with 4 distinct miRNAs (miR-378a, miR-379, miR-139, and miR-200) and sensitivity and specificity of 97.5% and 72%, respectively, and a diagnostic one characterized by the expression of 6 miRNAs (miR-151a, miR-30a-3p, miR-200b, miR-629, miR-100, and miR-154-3p) with 96% sensitivity and 60% specificity [66]. Yet another 8-miRNA signature was found, with miR-30b, miR-30c, miR-103, miR-122, miR-195, miR-203, miR-221, and miR-222 reported to be differentially expressed in exosomes isolated from NSCLC patients compared to healthy controls [67]. More recently, it was reported that an exosomal miRNA profile involving let-7b, let-7e, miR-23a-3p, and miR-486 was able to drastically discriminate lung cancer patients from healthy controls with an AUC of 0.899, specificity of 92.3%, and sensitivity of 80.3%. Interestingly, lung cancer histotypes were also distinguished by a 4-miRNA signature profile with good values of sensitivity and specificity [68]. Exosomal miR-96 was described as a diagnostic biomarker in NSCLC, and also a prognostic factor if correlated with tumor aggressiveness [69]. In resected lung squamous cell carcinoma, the levels of five miRNAs (miR-205, miR-19a, miR-19b, miR-30b, and miR-20a) identified inside exosomes were used as a diagnostic marker [70]. Exosomes also could be isolated from bronchoalveolar lavage or pleural effusion, and their miRNA cargo discriminated benign nodules from adenocarcinoma [74,75].

The miRNA load in exosomes was also utilized as a prognostic factor in lung cancer. The levels of miR-21 and miR-4257 levels in exosomes isolated from NSCLC patients with recurrence were highly comparable to patients without recurrence. These two exosomal miRNAs were also associated with tumor size, histotype, and metastasis [71]. Furthermore, a signature composed of 9 miRNAs was discovered and subsequently validated as a prognostic biomarker in lung cancer [72]. Exosomal miRNA (miR-25, miR-122, miR-195, miR-21, and miR-125b) modulation could also detect EGFR mutation and sensitivity to gefitinib in NSCLC patients, suggesting the potential of exosomes to select patients for target therapy [73]. Exosomal miR-221 and miR-222 presence was linked to a good response to osimertinib in EGFR-mutated lung cancer patients [18]. Recently, two clinical trials were approved to evaluate the safety of the efficacy of circulating exosomes and their non-coding RNA cargo for the diagnosis of lung cancer (NCT03542253; NCT03830619).

Growing evidence has reinforced the hypothesis that exosomes and, in particular, their miRNA cargo, have an essential impact on intercellular communication and that their identification could be employed as a promising diagnostic and/or prognostic biomarker in lung cancer detection.

All these studies highlight a major weakness for exo-miRNA research approaches that it is the lack of consensus on the isolation of vesicles. While some works utilize the magnetic beads methodology [64,65] others are in favor of commercially available kits or ultracentrifugation techniques, making it complicated to compare obtained results. Furthermore, limited number of NSCLC samples analyzed for each study with no additional validation on a larger cohort of individuals, makes the clinical utility of these biomarkers a bit fragile. On the other hand, all these studies make the point in demonstrating the efficacy as biomarkers for lung cancer diagnosis.

## 5. Circulating miRNAs in Exosomes: The bioMILD Experience

In our previous work, we focused on a cellular source of circulating miRNAs that are components of our MSC risk classifier, and observed several miRNAs not being released by non-epithelial cells (neither normal nor tumor cells) but, rather, by granulocytes and platelets, which represented a major contribution of miRNA release in blood [76]. These findings have supported our previous results that plasma MSC is independent from the molecular characteristics of tumor cells and detected to be positive even before a lesion is visible by LDCT [48,77]. Furthermore, the miRNA deregulation observed in the plasma of lung cancer patients was consistent with the modulation of the same miRNAs observed during the immunosuppressive conversion of immune cells. All these data imply that at least some circulating miRNAs may reflect a host-related signature rather than a tumor signature [76].

**Figure 2:**
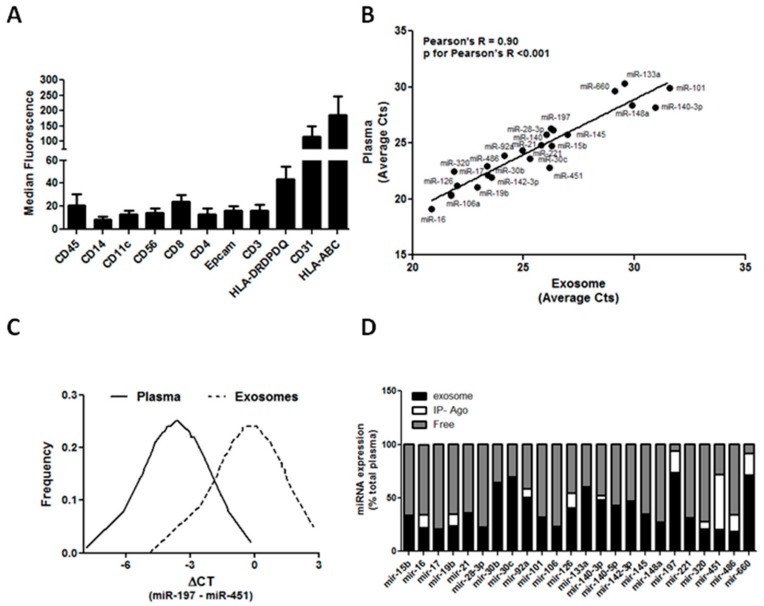
Exosomal miRNAs in the bioMILD trial. (**A**) Graphs show the surface markers on circulating exosomes in heavy smokers (*n* = 5). (**B**) Correlation of miRNA levels in plasma and in exosomes (*n* = 19 heavy-smokers volunteers and 8 lung cancer patients). (**C**) Normal distribution data for miR-197 and miR-451 in exosomes and plasma. (**D**) Physical status of circulating miRNAs in the plasma of heavy smokers (*n* = 5).

The expression of different exosomal surface markers, potential indicators of the cellular origin, was evaluated using a new multiplex bead-based flow cytometry method, Macsplex. Starting from 15 μg of total amount of protein of 5 cancer-free individuals, we observed that exosomes expressed several pan-hematopoietic markers, such as HLA and CD45. More specifically, circulating exosomes may be derived from specific blood cell subsets indicated by CD14, CD11c, CD56, and CD3 expression. Endothelial (CD31) and epithelial cell (Epcam) expression was also detected in exosomes isolated from these individuals (Figure 2A). These preliminary data suggest that circulating exosomes detected in plasma may reflect the activation/suppression of specific immune cells during lung carcinogenesis.

Circulating miRNAs are released by different cell components of the tumor and tumor microenvironment, mostly packed into exosomes or associated with protein complexes [78]. Concerning plasma samples, we first investigated whether differences in miRNA expression levels observed in the unfractionated plasma of patients and controls were maintained in the exosomal plasma fraction. For this, exosomes were isolated from 200 μL of plasma through Exoquick, and the miRNAs in exosomes together with their paired total plasma were assessed with custom microfluidic cards as previously described [79]. By analyzing the exosome fraction of 27 plasma samples of individuals with different plasma MSC risk profiles (19 heavy-smoker volunteers and 8 lung cancer patients), the presence of all the 24 miRNAs comprising the MSC within exosomes were confirmed. Moreover, by comparing miRNA CTs in plasma and their respective CTs in exosomes, a good degree of correlation (Pearson’s R = 0.90, *p* for Pearson’s R < 0.001) was observed, indicating that miRNA expression in exosomes could reflect miRNA levels in total plasma (Figure 2B). Interestingly, miR-133a, miR-197, miR-320, and miR-660 were enriched in exosome fractions, whereas miR-451 and miR-140-3p were present mostly outside exosomes.

Despite the good degree of correlation, the MSC test cannot be applied to the exosomes data [80]. However, given the normal distribution of data (Figure 2C), MSC cutoffs were scaled in order to maintain a fixed percentile of positive and negative cases. In this way, the MSC risk level using exosome or plasma data was concordant in 19 out of 27 (70%) patients (*p* = 0.002), thus resulting in a moderate inter-rater agreement with a Cohen’s kappa coefficient of 0.53.

In order to more precisely quantify the amount of miRNAs within exosomes, an adequate protocol was standardized for performing absolute quantification by digital PCR [81]. Moreover, a 24-miRNA analysis on total plasma, exosomes, and respective exosome-free plasma samples from 5 selected heavy-smoker individuals was carried out. Using a procedure based on published methods [82], the circulating miRNAs bound to Argonaute-2 (Ago2)—the most highly abundant protein that interacts with miRNAs—were immunoprecipitated in order to characterize circulating miRNA complexes in plasma. Interestingly, digital PCR data confirmed that miR-133, miR-197, and miR-660 were present mostly inside exosomes (with percentages inside exosomes of 60%, 73%, and 70%, respectively) suggesting a potential role for these specific miRNAs in lung tumorigenesis [83,84]. Furthermore, our data showed that most miRNAs are quite similarly distributed inside and outside exosomes, whereas the remaining ones, such as the hemolysis-related miRNAs miR-16, miR-451, and miR-486, are mostly bound to Ago2, in keeping with their release through blood cell lysis (Figure 2D).

## 6. Conclusions

The tumor microenvironment exerts a key role in lung carcinogenesis [13] and, in particular, extracellular vesicles show the ability to modulate the phenotype of the microenvironment cells that directly surround the primary tumor or the metastatic niche [85]. Several cellular mechanisms were affected by exosomes, such as oncogenic transfer, angiogenesis, and pre-metastatic niche formation [86]. Recent data on exosomes has strongly suggest that these extracellular vesicles could represent an original class of diagnostic and predictive biomarkers for minimally invasive liquid biopsy [87]. Exosomes could be taken up by neighboring or distant cells and are capable of modulating the functionality of recipient cells through the transfer of a variety of information, such as proteins, lipids, RNAs, and miRNAs [24]. It has been demonstrated that tumor cells release exosomes in body fluids and that these vesicles alter the surrounding microenvironment in such a way that it facilitates and supports tumor growth and metastasis [88]. As described in this review, exosomal miRNAs demonstrate good sensitivity and specificity as a result of their stability, and have great potential to become part of a routine laboratory test in the near future.

Minimally invasive circulating biomarkers research, as intended as miRNAs, exosomes and exo-miRNAs, is yet at an early phase, therefore it is critical that all challenging issues that complicate their clinical application in lung cancer detection are delineated and solved. An adequate isolation and purity of exosomes preparation is the most important aspect in order to obtain the most reliable results; presently the method of choice is ultracentrifugation that, however, appears to be time consuming and complicated by the presence of protein or other extracellular vesicles precipitation, which reduces and alters exosomes pureness. Also, several studies highlighted concerns such as poor reproducibility and high heterogeneity attributed to the different origin of circulating miRNAs, but also to methodological aspects of miRNAs analysis and data evaluation [89].

Other factors that should be taken in consideration are the mechanisms behind exosomes biogenesis and, in particular, the uptake from recipient cells should be better clarified and investigated so to generate and develop more efficient diagnostic biomarkers. In addition, determinants such as physical activity and time of sample collection could influence the levels of circulating vesicles. It is important to establish and define consolidated and standard operating procedures to improve reproducibility and accuracy of these specific non-invasive circulating biomarkers studies so to make the transition in the clinical environment as biomarkers for the diagnosis of lung cancer, an easier and more efficient step. Even if the advances in technologies and bioinformatic tools will certainly contribute to the transfer of more robust assays into the clinical practice, nonetheless we must re-think the idea of a single biomarker standing alone. Ongoing and future studies plan to combine epidemiological factors, radiomics signatures and blood biomarkers using artificial intelligence approaches to improve risk prediction models, implement the accuracy and improve the benefit of lung cancer screening. We afforded this challenge and in 2013 we launched a screening trial, bioMILD (NCT02247453) were we combine LDCT and blood miRNAs as forefront tests in a large prospective screening trial of 4119 smokers in the attempt to provide the best algorithm to improve accuracy of lung cancer risk prediction, differential diagnosis of indeterminate LDCT lesions as well to personalize screening intervals. These new data, together with those deriving from the analysis of the performance of miRNA test in the long-term mortality risk of the MILD trial [47] will provide an important confirmation of the clinical utility of miRNAs biomarkers in lung cancer screening.

Finally, the role of exosomes as therapeutic agents should be thoroughly considered in the future. Exosomes have a lipid bilayer structure with low immunogenicity and toxicity, and are not be recognized by the mononuclear phagocyte system. Due to these peculiar characteristics, loading exosomes with molecules such as miRNA, siRNA, and synthetic small-molecule drugs could be a novel therapeutic strategy for lung cancer. To date, there are only a few studies describing attempts to load drugs into extracellular vesicles and demonstrate the anti-proliferative effects of these compounds in lung cancer models [90,91]. However, a large amount of work should be done to elucidate the promising roles of exosomes as therapeutic agents for lung cancer treatment.

In conclusion, this review summarizes the current state-of-the-art of exosomes and, in particular, their miRNA cargo for liquid biopsy in lung cancer. Additional studies are needed to better characterize the role of exosomes in lung cancer, but they can already be considered promising biomarkers for clinical use.

## Figures and Tables

**Table 1:** Circulating miRNAs for lung cancer detection.

miRNA Signature	Specimen	Performance	Patients Enrolled	Ref.
miR-29/miR-146b/miR-221/let-7a/miR-155/miR-17/miR-27a/miR-106a	Serum	AUC ^1^ of 0.60	220 cases (Stage I–II), 220 Controls	[36]
miR-21/miR-126/miR-210/miR-486	Plasma	86.2% sensitivity 96.6% specificity	58 cases (30 Stage I–II, 28 Stage III–IV), 29 Controls	[37]
miR-155/miR-197/miR-182	Plasma	81.3% sensitivity 86.8% specificity	74 cases (33 Stage I–II, 41 Stage III–IV), 68 Controls	[38]
miR-483/miR-193a-3p/miR-25/miR-214/miR-7	Serum	AUC of 0.979	221 cases (83 Stage I–II, 130 Stage III–IV, 8 NA), 161 Controls	[39]
24 miRNAs	Plasma	87% sensitivity 81% specificity	69 cases (37 Stage I, 12 Stage II–III, 19 Stage IV), 870 Controls	[40]
13 miRNAs	Serum	77.8% sensitivity 74.8% specificity	74 cases (42 Stage I–II, 32 Stage II–III), 1115 Controls	[41]
24 miRNAs	Plasma	AUC of 0.92	100 cases (42 Stage I–II–III), 100 Controls	[42]
miR-21/miR-223/miR-155/miR-126	Serum	83% sensitivity 84% specificity	6919 cases, 7064 Controls	[43]

^1^ AUC: area under the curve.

**Table 2:** Relevant studies on tumor exosomes and their functional role in lung cancer.

Exosomal-miRNA	Target	Function	Ref.
miR-21/miR-29a	TLRs	Support lung tumor growth and metastasis	[33]
miR-23	PHD, ZO-1	Enhance angiogenesis and vascular permeability	[52]
miR-103	PTEN	Promote tumor progression and angiogenesis	[21]
miR-210	TIMP1	Increase angiogenesis and tumor progression	[53]
miR-494/miR-542-3p	cadherin-17	Modulate pre-metastatic niche	[54]
miR-512/miR-373	TEAD4, RelA, PIK3CA	Cisplatin sensitivity and suppression of tumor growth	[55]
miR-23	CD107	Inhibit NK cell function	[56]
miR-302b	TGFBRII	Block proliferation and migration	[57]
miR-9	SOCS5	Promote endothelial migration and angiogenesis	[58]
miR-21	STAT3	Promote angiogenesis and tumor growth	[59]

TLRs: Toll-like receptors; PHD: prolyl hydroxylase; ZO-1: zonula occludens-1; PTEN: phosphatase and tensin homolog; TIMP1: Tissue inhibitor of metalloproteinase 1; TGFBRII: transforming growth factor beta receptor II; SOCS5 suppressor of cytokine signaling 5.

**Table 3:** Exosomal miRNAs studies in lung cancer.

miRNAs	Specimen	Clinical significance	Ref.
miR-17-3p/miR-21/miR-106a/miR-146/miR-155/miR-191/miR-192/miR-203/miR-205/miR-210/miR-212/miR-214	Plasma	Diagnostic	[64]
let-7f/miR-20b/miR-30e-3p/miR-223/miR-301	Plasma	Diagnostic/prognostic	[65]
miR-378a/miR-379/miR-139/miR-200	Plasma	Diagnostic	[66]
miR-151a/miR-30a-3p/miR-200b/miR-629/miR-100/miR-154-3p	Plasma	Diagnostic	[66]
miR-30b/miR-30c/miR-103/miR-122/miR-195/miR-203/miR-221/miR-222	Plasma	Diagnostic	[67]
let-7b/let-7e/miR-23a-3p/miR-486	Plasma	Diagnostic	[68]
miR-96	Plasma	Diagnostic	[69]
miR-205/miR-19a/miR-19b/miR-30b/miR-20a	Plasma	Diagnostic	[70]
miR-21/miR-4257	Plasma	Prognostic/predictor	[71]
miR-23b-3p/miR-10b/miR-21	Plasma	Prognostic	[72]
miR-25/miR-122/miR-195/miR-21/miR-125b	Plasma	Prognostic	[73]
miR-221 and miR-222	Plasma	Predictor of response	[18]
